# Supplementary material for: The development and evolution of inhibitory neurons in primate cerebrum
Source: Nature. 2022 Mar 23;603(7903):871–7. doi: 10.1038/s41586-022-04510-w (PMC8967711; doi:10.1038/s41586-022-04510-w)
Supplement: Supplementary file 1 — Reporting Summary [file 41586_2022_4510_MOESM1_ESM.pdf]

nature research

Corresponding author(s): Alex A. Pollen
Last updated by author(s): Nov 29, 2021

Reporting Summary

Nature Research wishes to improve the reproducibility of the work that we publish. This form provides structure for consistency and transparency in reporting. For further information on Nature Research policies, see our Editorial Policies and the Editorial Policy Checklist.

Statistics

For all statistical analyses, confirm that the following items are present in the figure legend, table legend, main text, or Methods section.

n/a	Confirmed	
☐	☒	The exact sample size (*n*) for each experimental group/condition, given as a discrete number and unit of measurement
☐	☒	A statement on whether measurements were taken from distinct samples or whether the same sample was measured repeatedly
☐	☒	The statistical test(s) used AND whether they are one- or two-sided<br>*Only common tests should be described solely by name; describe more complex techniques in the Methods section.*
☐	☒	A description of all covariates tested
☐	☒	A description of any assumptions or corrections, such as tests of normality and adjustment for multiple comparisons
☐	☒	A full description of the statistical parameters including central tendency (e.g. means) or other basic estimates (e.g. regression coefficient) AND variation (e.g. standard deviation) or associated estimates of uncertainty (e.g. confidence intervals)
☐	☒	For null hypothesis testing, the test statistic (e.g. *F*, *t*, *r*) with confidence intervals, effect sizes, degrees of freedom and *P* value noted<br>*Give P values as exact values whenever suitable.*
☒	☐	For Bayesian analysis, information on the choice of priors and Markov chain Monte Carlo settings
☒	☐	For hierarchical and complex designs, identification of the appropriate level for tests and full reporting of outcomes
☐	☒	Estimates of effect sizes (e.g. Cohen's *d*, Pearson's *r*), indicating how they were calculated

Our web collection on statistics for biologists contains articles on many of the points above.

Software and code

Policy information about availability of computer code

Data collection

In generating data, software used included Thermo Fisher Evos M7000 microscope control software, Illumina sequencer control software and bcl2fastq (bcl2fastq2) software.

Data analysis

Single cell RNA-seq analysis was carried out in custom python and bash scripts, implementing published algorithms as described and cited in methods using published algorithms.

Quality control: Kallisto-Bus output matrix files (including both introns and exons together) were input to Cellbender (release 0.2.0, https://github.com/broadinstitute/CellBender), which was used to remove likely ambient RNA only. Only droplets with greater than 0.99 probability of being cells (not ambient RNA), calculated by the Cellbender model, were included in further analysis. Droplets with fewer than 800 genes detected, or greater than 40% ribosomal or 15% mitochondrial reads were filtered from the dataset. Doublets were then detected and removed from the dataset using scrublet (release 0.2.2, using threshold parameter 0.5).

Clustering and determining homologous cell types: Much of the analysis pipeline is based on scanpy infrastructure and anndata data structures48. Counts in cells were normalized by read depth, log transformed and then scaled for each gene across all cells. Principal component analysis was then performed using the top 12,000 most variable genes (using the original Seurat variable genes selection method, implemented in the scanpy package), with the 100 most variance-encompassing principal components being used for the following steps. Batch correction was limited to the requirement that highly variable genes be variable in more than one sequencing sample and by application of batch-balanced k-nearest neighbors (BBKNN)49, using Euclidean distance of principal components to find 3 neighbors per batch in the developing data, and 12 neighbors per dataset in the developing and adult merged mouse data. Using BBKNN-derived k-nearest neighbors graphs, Leiden clustering was then applied to cluster based upon the KNN graph with scanpy's resolution parameter set to 10 (or 7 in the developing mouse dataset). Glia, along with excitatory progenitor and neuron clusters were removed from the dataset in non-ganglionic eminence batches if they had below mean expression value for two or more GAD1/2 and DLX1/2/5/6 genes, with Cajal-Retzius cells (RMTW_ZIC1/RELN) meeting this threshold and serving as a useful out-group (these cells were called RMTW-derived based on the ZIC1/2 and RELN expression, though they are known to have multiple origins50). Following removal of non-INs, scaling, PCA and the following steps were

repeated with this final IN dataset.

High-resolution Leiden clusters partitioned continuous differentiation trajectories of post-mitotic initial classes into subclusters based on maturation stage. These high resolution clusters were then merged to initial classes manually, using hierarchical clustering of cluster gene expression averages and distinctness of individual Leiden cluster markers as a guide. The nomenclature for merged clusters incorporates the presumptive spatial origin of initial classes and specific marker genes. Spatial origin for each class was inferred based on the expression of canonical marker genes for RMTW, MGE, LGE, CGE, and VMF (e.g., LHX5, NKX2.1, MEIS2, NR2F2, ZIC1) and supported by immunostaining and by the enrichment of these genes in cells from region-specific dissections. For merged species analysis, genes were normalized and scaled within species, then merged for downstream analysis using BBKNN (with 25 neighbors across and within species, with the mutual nearest neighbors used for the Sankey plot comparison of developing macaque and developing mouse). Following clustering, mean expression in each class was calculated for each gene which was among the original 12,000 most variable 1-to-1 orthologs from each dataset that were variable in both species (6,227 genes). These classes were then compared across species by Pearson correlation of their gene expression vectors.

Trajectory analysis of activating and inactivating macaque genes: We applied scVelo's dynamical model (release 0.2.3) 51 to derive a shared latent time based on RNA velocity using spliced and unspliced counts from kallisto. Next, we used the related cellrank (release 1.3.1) package15 to derive absorption probabilities of immature cells in the "Transition" cluster to likely initial classes.

Image processing: Stitching was carried out using python scripts implementing ImageJ's max correlation Grid/Collection stitching (V1.2). Downstream image background subtraction, brightness/contrast adjustment, merging and montaging was also performed using ImageJ (1.53c).

For manuscripts utilizing custom algorithms or software that are central to the research but not yet described in published literature, software must be made available to editors and reviewers. We strongly encourage code deposition in a community repository (e.g. GitHub). See the Nature Research guidelines for submitting code & software for further information.

Data

Policy information about availability of data

All manuscripts must include a data availability statement. This statement should provide the following information, where applicable:

- Accession codes, unique identifiers, or web links for publicly available datasets
- A list of figures that have associated raw data
- A description of any restrictions on data availability

The sequencing data have been deposited in GEO under the accession GSE169122, and the data is browsable at https://cells-test.gi.ucsc.edu/?ds=macaque-dev-inhibitory-neurons. Python scripts to analyze data and generate figures are saved on github and are available upon request. Raw imaging data are available on request, though we are working to make all files available. The images are currently too large, numerous and complex

Field-specific reporting

Please select the one below that is the best fit for your research. If you are not sure, read the appropriate sections before making your selection.

☒ Life sciences ☐ Behavioural & social sciences ☐ Ecological, evolutionary & environmental sciences

For a reference copy of the document with all sections, see nature.com/documents/nr-reporting-summary-flat.pdf

Life sciences study design

All studies must disclose on these points even when the disclosure is negative.

Sample size	Data was collected from as many individuals as were available, with 9 embryos being collected and an additional 2 from public data being utilized. No sample size calculation was performed, as we do not attempt to test individual-level covariates (individual, sex, timepoint).
Data exclusions	Excitatory neurons and their progenitors, as well as glia were filtered from the dataset bioinformatically. This has been done in previous Nature studies, for example Krienen et al., 2020.
Replication	In order to improve replicability, samples were collected across 3 years from 9 different individuals. Findings were further verified against data from public datasets, as well as in another species (mouse). Furthermore, immunofluorescence microscopy was used to corroborate findings in at least one and often multiple different brains. This is above the standard of similar studies of this type published in major journals at this time.
Randomization	Randomization was not relevant to this study.
Blinding	Blinding was not relevant to this study.

Reporting for specific materials, systems and methods

We require information from authors about some types of materials, experimental systems and methods used in many studies. Here, indicate whether each material, system or method listed is relevant to your study. If you are not sure if a list item applies to your research, read the appropriate section before selecting a response.

Materials & experimental systems

n/a	Involved in the study
☐	☒ Antibodies
☒	☐ Eukaryotic cell lines
☒	☐ Palaeontology and archaeology
☐	☒ Animals and other organisms
☐	☒ Human research participants
☒	☐ Clinical data
☒	☐ Dual use research of concern

Methods

n/a	Involved in the study
☒	☐ ChIP-seq
☒	☐ Flow cytometry
☒	☐ MRI-based neuroimaging

Antibodies

Antibodies used

PAX6
Rabbit
BioLegend
901301
1:750
lot: B277104

PAX6
Sheep
R&D
AF8150
1:200
lot:CDJL0420021

NKX2-1
Rabbit
Millipore
MILL-07-601
1:750
lot: I3448599

FOXP2
Mouse
Millipore
MABE415
1:500
lot: 3278590
FOXP2
Sheep
R&D
AF5647
1:2000

FOXP4
Rabbit
Millipore
ABE74
1:500
lot: 3050680
NR2F2
Mouse
Perseus Proteomics
PP-H7147-00
1:750
lot: A-2

SCGN
Goat
Fisher
AF4878SP
1:500
Lot CASX0119111

MEIS2
Mouse
Sigma
WH0004212M1

1:500
lot: J1071-1H4

SP8
Rabbit
Millipore
HPA054006
1:500
lot: 000003237

TH
Sheep
Millipore
AB1542
1:1000
lot: 3403416

TH
Rabbit
Millipore
AB152
1:1000
lot: 3574360

MKI67
Mouse
Dako
MIB-1
1:250
20057589

Validation

Antibodies are all common and commercially available and have been widely used. Antibodies were used on large tissues, and for each one, the entire section. Each antibody is thus internally controlled by expected anatomical distribution and subcellular localization.

Animals and other organisms

Policy information about studies involving animals; ARRIVE guidelines recommended for reporting animal research

Laboratory animals

Postmortem macaque embryonic brain tissue (between embryonic day 40 to 100) of unknown sex was obtained from the California National Primate Research Center at UC-Davis.

Wild animals

Provide details on animals observed in or captured in the field; report species, sex and age where possible. Describe how animals were caught and transported and what happened to captive animals after the study (if killed, explain why and describe method; if released, say where and when) OR state that the study did not involve wild animals.

Field-collected samples

For laboratory work with field-collected samples, describe all relevant parameters such as housing, maintenance, temperature, photoperiod and end-of-experiment protocol OR state that the study did not involve samples collected from the field.

Ethics oversight

Macaque tissue was generously provided by the UC Davis Primate Center. All animal procedures conformed to the requirements of the Animal Welfare Act and protocols were approved prior to implementation by the Institutional Animal Care and Use Committee (IACUC) at the University of California, Davis. De-identified tissue samples were collected with previous patient consent in strict observance of the legal and institutional ethical regulations. Protocols and samples were approved by UCSF GESCR (Gamete, Embryo, and Stem Cell Research) Committee.

Note that full information on the approval of the study protocol must also be provided in the manuscript.

Human research participants

Policy information about studies involving human research participants

Population characteristics

N/A

Recruitment

N/A

Ethics oversight

Protocols were approved by the Human Gamete, Embryo and Stem Cell Research Committee (institutional review board) at the University of California, San Francisco

Note that full information on the approval of the study protocol must also be provided in the manuscript.
